# Supplementary material for: The Immobilization of a Cyclodipeptide Synthase Enables Biocatalysis for Cyclodipeptide Production
Source: ACS Sustain Chem Eng. 2024 Aug 22;12(35):13080–9. doi: 10.1021/acssuschemeng.4c01230 (PMC11372833; doi:10.1021/acssuschemeng.4c01230)
Supplement: Supplementary file 1 — sc4c01230_si_001.pdf [file sc4c01230_si_001.pdf]

Supporting information for "**Overcoming the challenges of cascade reactions and complex substrates in natural product biocatalysis: Immobilization of a cyclodipeptide synthase**."

Lynette Alvarado-Ramírez[a], Emmajay Sutherland[b], Elda M. Melchor-Martíneza[c], Roberto Parra-Saldívara[c], Alfredo D. Bonaccorso[d], Clarissa Melo Czekster[b]*

[a] School of Engineering and Sciences, Tecnologico de Monterrey, Monterrey 64849, Mexico.
[b] School of Biology, University of St Andrews, North Haugh, St Andrews, KY16 9ST, UK.
[c] Institute of Advanced Materials for Sustainable Manufacturing, Tecnologico de Monterrey, Monterrey 64849, Mexico
[d] School of Chemistry, University of St Andrews, North Haugh, St Andrews, KY16 9ST, UK.

*Corresponding author email: **cmc27@st-andrews.ac.uk**

Supporting Figures 3

Figure S1. FTIR spectra of biochar samples from spent coffee (a), distillery waste (b), plastic & wood waste (c), macroalgae (d), Ca-alginate beads €, alginate coated beads (f), chitosan beads (g). 3

Figure S2. HPLC chromatogram of the purification of cHE at 214 nm and calibration curve used a for the quantification of the cHE. Structure of the cHE was showed. 4

Figure S3. (a) Effect of PbCDPS loading in the immobilization in alginate beads, and ratio of PbCDPS/cHE). (b) Effect of PbCDPS loading in the immobilization on biochar from spent coffee, and ratio of PbCDPS/cHE. 4

Figure S4. RNA denaturing gel. The identification of the lanes are as follows: 1-molecular weight marker; 2-supernatant after reaction with $PbCDPSbiochar\ spent\ coffeeglut$; **3-supernatant after reaction with** $PbCDPSalg\ beads$; **4-supernatant after reaction with** $PbCDPSchi\ beadsglut$; **5-**$PbCDPSbiochar\ spent\ coffeeglut$ **support from; 6-**$PbCDPSalg\ beads$; **support after reaction; 7-**$PbCDPSchi\ beadsglut$**support after the reaction; 8-molecular weight marker; 9-tRNA pool.** 5

Figure S5. SDS-PAGE for immobilization mixture loaded with 20 μM total protein per lane. The identification of the lanes are as follows: 1- molecular weight ladder; 2-PbCDPS; 3- HisRS; 4-GluRS; 5- supernatant after reaction with $PbCDPSchi\ beadsglut$; **6-supernatant after reaction with** $PbCDPSalg\ beads$; **7-supernantat after reaction with** $PbCDPSbiochar\ spent\ coffeeglut$; **8-**$PbCDPSchi\ beadsglut$**support after reaction; 9-**$PbCDPSalg\ beads$ **support after reaction; 10- support from** $PbCDPSbiochar\ spent\ coffeeglut$. 6

Figure S6. Effect of different quenching strategies for the measurement of cHE the selected supports (PbCDPSalg beads,PbCDPSchi beadsglut**and,** PbCDPSbiochar spent coffeeglut**)** 6

Supporting Tables 7

Table S1. BET specific surface area, pore volume and pore size of biochar from macroalgae, spent coffee, distillery waste and plastic & wood waste with and without activation with CO_2. 7

Table S2. Immobilization of PbCDPS on activated carbon and cHE product formation. 7

Table S3. Loading effect of PbCDPS on the immobilization of chitosan activated beads. 7

Table S4. Co-immobilization of HisRS and Glu-RS in alginate beads, chitosan beads activated with glutaraldehyde and biochar from spent coffee activated with glutaraldehyde. 8

Supporting Figures

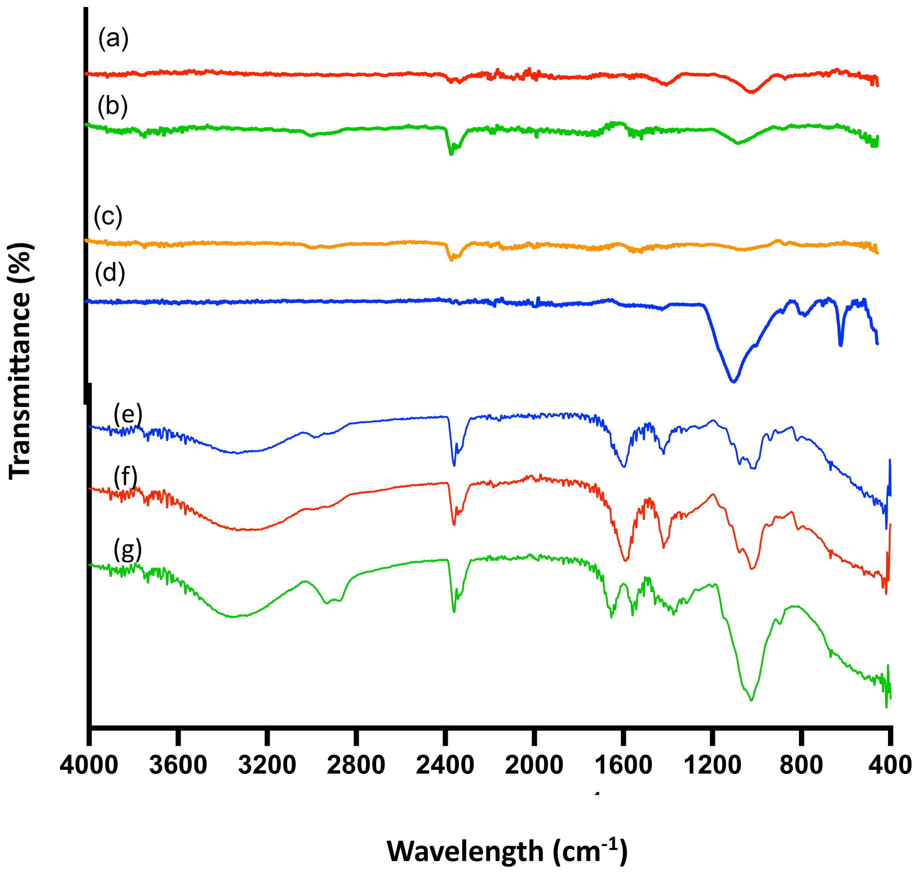

Figure S1. FTIR spectra of biochar samples from spent coffee (a), distillery waste (b), plastic & wood waste (c), macroalgae (d), Ca-alginate beads €, alginate coated beads (f), chitosan beads (g).

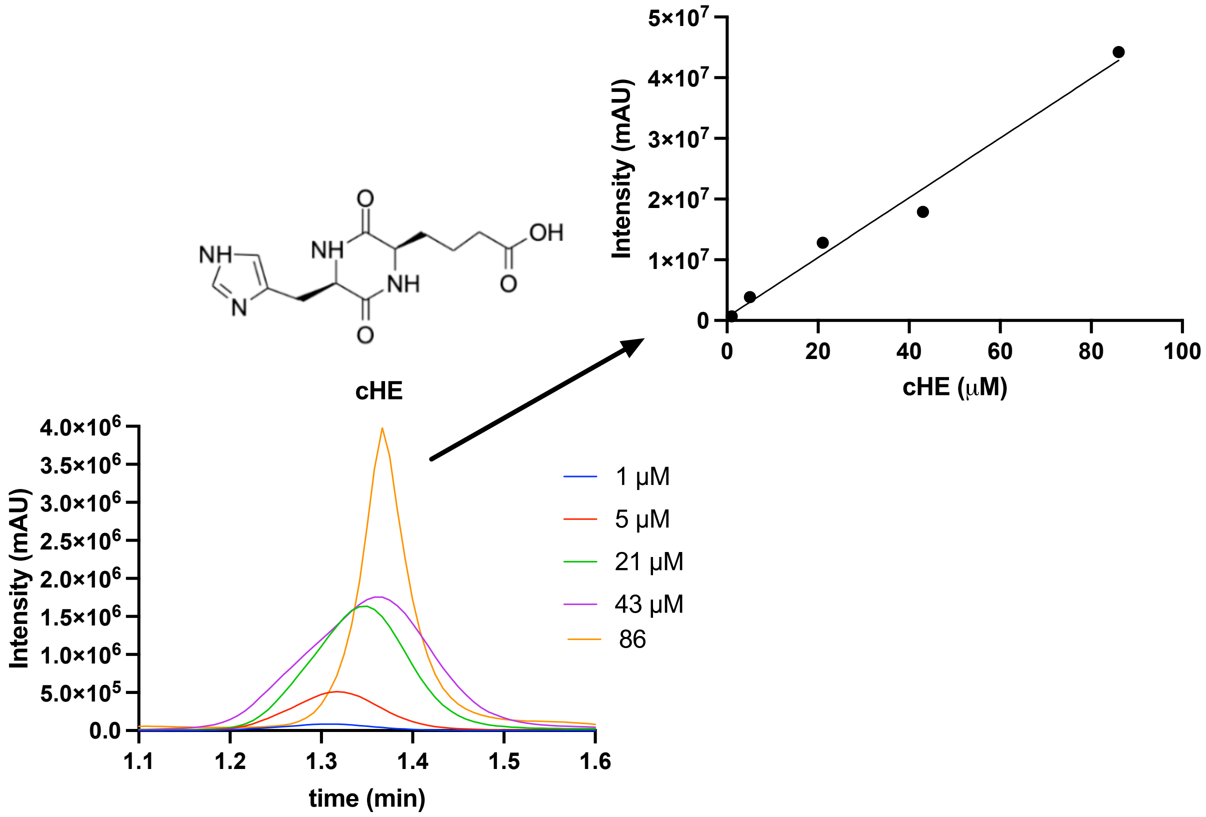

Figure S2. HPLC chromatogram of the purification of cHE at 214 nm and calibration curve used a for the quantification of the cHE. Structure of the cHE was showed.

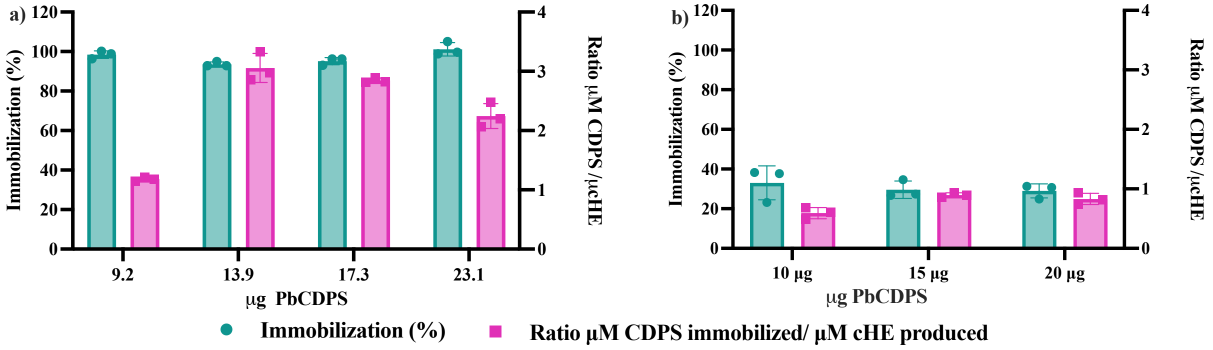

Figure S3. (a) Effect of PbCDPS loading in the immobilization in alginate beads, and ratio of PbCDPS/cHE). (b) Effect of PbCDPS loading in the immobilization on biochar from spent coffee, and ratio of PbCDPS/cHE.

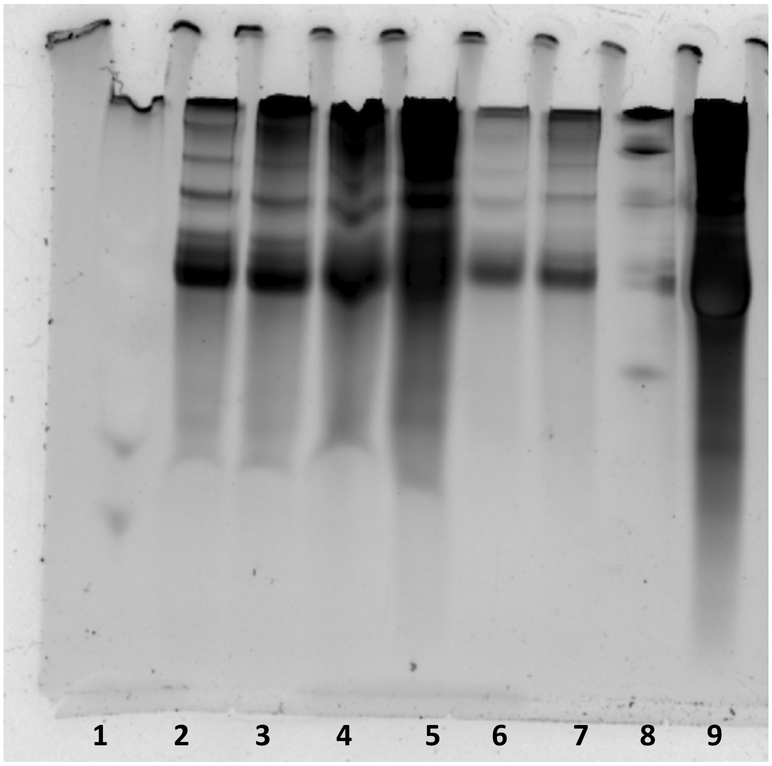

Figure S4. RNA denaturing gel. The identification of the lanes are as follows: 1-molecular weight marker; 2-supernatant after reaction with $PbCDPS^{glut}_{biochar\ spent\ coffee}$; 3-supernatant after reaction with $PbCDPS_{alg\ beads}$; 4-supernatant after reaction with $PbCDPS^{glut}_{chi\ beads}$; 5-$PbCDPS^{glut}_{biochar\ spent\ coffee}$ support from; 6-$PbCDPS_{alg\ beads}$; support after reaction; 7- $PbCDPS^{glut}_{chi\ beads}$support after the reaction; 8-molecular weight marker; 9-tRNA pool.

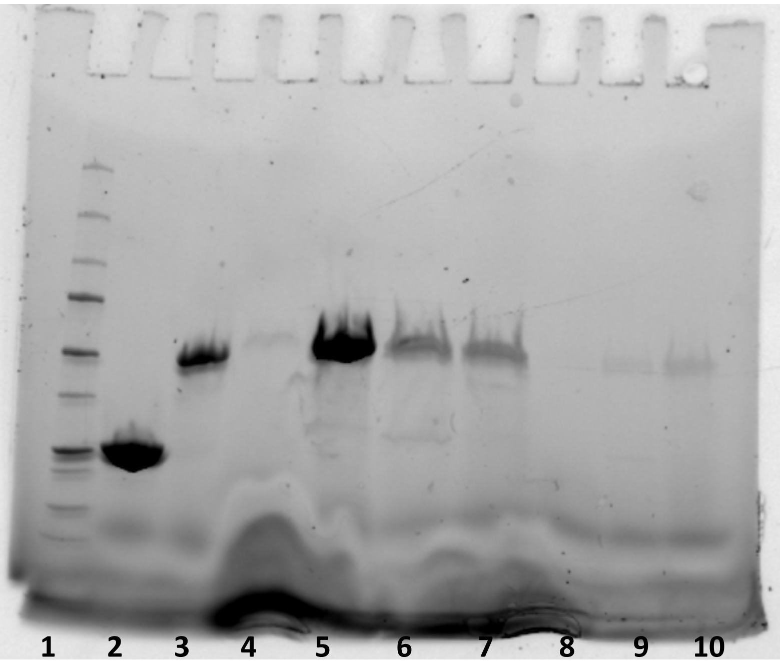

Figure S5. SDS-PAGE for immobilization mixture loaded with 20 μM total protein per lane. The identification of the lanes are as follows: 1- molecular weight ladder; 2- PbCDPS; 3- HisRS; 4-GluRS; 5- supernatant after reaction with $PbCDPS^{glut}_{chi\ beads}$; 6- supernatant after reaction with $PbCDPS_{alg\ beads}$; 7-supernantat after reaction with $PbCDPS^{glut}_{biochar\ spent\ coffee}$; 8-$PbCDPS^{glut}_{chi\ beads}$support after reaction; 9- $PbCDPS_{alg\ beads}$ support after reaction; 10- support from $PbCDPS^{glut}_{biochar\ spent\ coffee}$.

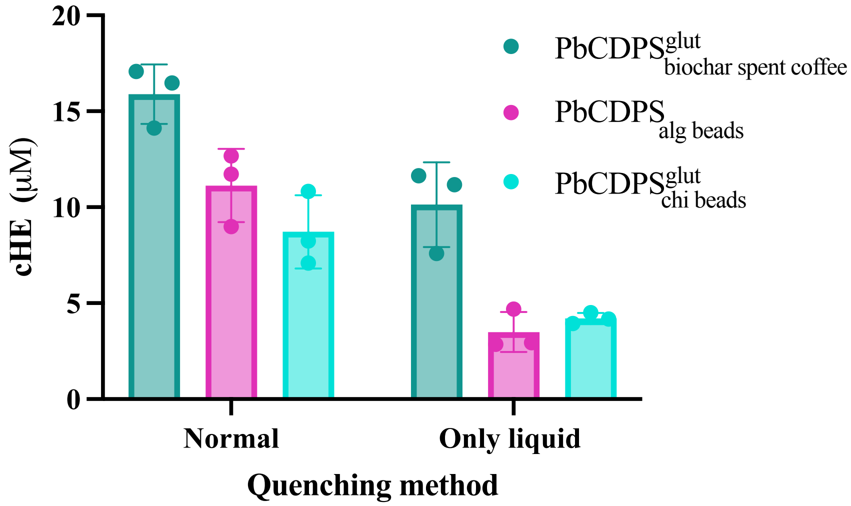

Figure S6. Effect of different quenching strategies for the measurement of cHE the selected supports ($\mathrm{PbCDPS_{alg\ beads}}$,$\mathrm{PbCDPS^{glut}_{chi\ beads}}$and, $\mathrm{PbCDPS^{glut}_{biochar\ spent\ coffee}}$)

Supporting Tables

Table S1. BET specific surface area, pore volume and pore size of biochar from macroalgae, spent coffee, distillery waste and plastic & wood waste with and without activation with CO_2.

**Support**	**Surface area (m^2/g)**	**Pore volume (cc/g)**	**Pore size (nm)**
Macroalgae	73.09	0.092	1.641
Macroalgae CO_2	46.92	0.126	0.017
Spent coffee	3.738	0.005	3.791
Spent coffee CO_2	14.41	0.026	0.014
Distillery waste	6.793	0.011	2.769
Plastic & wood waste	17.81	0.003	2.769
Plastic & wood waste CO_2	18.11	0.3867	0.0101

Table S2. Immobilization of PbCDPS on activated carbon and cHE product formation.

**Time (h)**	**Glutaraldehyde concentration (%)**	**Immobilization (%)**	**cHE (mM)**
0.5		23.5	2.72
1	1	22.2	ND
20		48.9	3.43
0.5		14.8	5.23
1	2	14	ND
20		45.8	7.59

ND: not determined as HPLC UV peak area is indistinguishable from noise.

Table S3. Loading effect of PbCDPS on the immobilization of chitosan activated beads.

**PbCDPS (μg)**	**Immobilization (%)**
7.3	84.58 ± 3.95
14.6	83.67 ± 4.66
29.2	87.44 ± 1.97
58.4	86.87 ± 1.78

Table S4. Co-immobilization of HisRS and Glu-RS in alginate beads, chitosan beads activated with glutaraldehyde and biochar from spent coffee activated with glutaraldehyde.

System	Immobilization (%)
$PbCDPS_{alg\ beads}$	100 ± 1.15
$PbCDPS_{chi\ beads}^{glut}$	52.48 ± 9.10
$PbCDPS_{biochar\ spent\ coffee}^{glut}$	24.47 ± 7.99
